# From online to offline education in the post-pandemic era: Challenges encountered by international students at British universities

**DOI:** 10.3389/fpsyg.2022.1093475

**Published:** 2023-01-18

**Authors:** Xin Zhao, Wenchao Xue

**Affiliations:** Information School, University of Sheffield, Sheffield, United Kingdom

**Keywords:** online-offline transition, international student experience, post-pandemic, student transition, learning experience

## Abstract

**Background:**

After 2 years of anti-pandemic struggles, universities in the United Kingdom have started to witness a reverse transition, a shift from online to offline education. This includes encouraging students to begin face-to-face programmes and allowing flexibility for remote learners, but later requiring all students to return to campus by a certain date.

**Objectives:**

This paper aims to explore the challenges and impacts brought about by this new transition and provide recommendations for universities to enhance student experience for future adversity.

**Method:**

This qualitative study conducted semi-structured interviews with 24 international students from a British university to explore their experiences during the transition. The results were analysed using thematic analysis.

**Results:**

Our data revealed both internal and external challenges to students during the online-to-offline shift, which lead to a general resistance to said shift. Specifically, policy challenges (e.g., policy conflicts) imposed the most significant impacts on international students, resulting in psychological anxiety, financial losses, and negative learning experiences. The reduction of digital tools and learning materials during the shift also presented challenges to students who developed a reliance on digital resources while learning remotely. Other challenges have also been identified, including academic barriers and social engagement issues.

**Conclusion:**

By highlighting these challenges, this paper has practical implications for university policy decisions and provides recommendations for supporting students’ transition back to traditional offline learning.

## 1. Introduction

Worldwide educational institutions have been affected by the outbreak of the COVID-19 pandemic. According to a report from the United Nations Educational, Scientific and Cultural Organization (UNESCO, 2021), over 220 million students were impacted by this unprecedented incident. The impact included social-distancing measures, lecture cancellations and teaching facility closures (Marinoni et al., 2020). Consequently, campus-based education activities were moved to online or hybrid environments, creating various challenges for students (Lemay et al., 2021). After over 2 years of anti-pandemic struggles, many countries have implemented less severe public health and social measures (WHO, 2022). Since the summer of 2021, the UK government announced its anti-COVID strategy, ‘Living with COVID-19’ (Cabinet Office, 2022). Following this national policy, the Department of Education of the United Kingdom started to encourage higher education institutions to return to delivering face-to-face teaching (Department of Education, 2022). As a result, most universities in the United Kingdom switched to face-to-face teaching, making students readjust to a reverse transition from online to offline learning, causing new challenges for students, especially those with international backgrounds. However, most research on student transitions related to the pandemic and post-pandemic education has focused on challenges during online transitions (Saikat et al., 2021; Gupta et al., 2022; Szopiński and Bachnik, 2022). A limited amount of research has been conducted on the reverse transition, in which students switched from online to offline learning.

This research aims to examine the experiences of Chinese international students during the post-pandemic transition from online to offline learning and asks the following questions:

- What challenges and constraints do Chinese international students face during the online-offline transition?
- What suggestions and improvements can be made to enhance the student experience for future adversity?

The project used a research university (S University) in the United Kingdom as an example before exploring the impact of the online-to-offline transition on a group of Chinese international students (*n* = 24). During the first half of the academic term, S University adopted a hybrid teaching approach, encouraging students to return to campus for face-to-face teaching while providing online alternatives for those temporarily based outside of the United Kingdom. Once students arrived on campus, they were required to attend face-to-face classes. During the second half of the academic term, S University cancelled all online teaching and completely shifted their courses offline. Students who could not reach the campus on time were required to take a Leave of Absence (LOA) and postpone their studies for a year. Depending on when these students arrive on campus, they may face different challenges during this reverse transition. Accordingly, this research defines the following student groups:

- Campus Starter (CS): Students who arrived at the university before the term started and attended the face-to-face courses (as encouraged).
- Remote Starter (RS): Students who arrived at the university after a period of distance learning and attended face-to-face courses before the start of the second half of the academic term.
- Leave of Absence Students (LOA): Students who completed the first half of the academic term remotely, but could not arrive at the university in time for the second half of the academic year for in-person teaching. They were placed on ‘leave of absence’ and had to postpone their studies for a year.

## 2. Literature review

### 2.1. Issues associated with online teaching during the pandemic

As a preventive measure to curb the spread of the coronavirus, strict social restrictions have been adopted by many countries, such as keeping in place social distancing, remote work, and regional lockdowns (Kaur et al., 2021). As a result, educational institutions around the world have been heavily impacted and forced to adopt a distance-learning approach (Gillis and Krull, 2020; Lederman, 2020; Zhao et al., 2020). However, research suggests that there are many concerns associated with the sudden shift to online teaching, such as technical issues, student disengagement, and time zone differences, which may cause social and technical challenges for both teachers and students (Fatoni et al., 2020; Lemay et al., 2021; Naddeo et al., 2021). For example, Daniel (2020) argues that instructors were unprepared for online teaching, due to a lack of training and support from universities. Naddeo et al. (2021) found that students’ negativity is related to Internet connectivity and the performance of learning management systems (LMS). Similarly, Song et al. (2004) found that, in addition to connectivity issues, the students’ experience can be negatively influenced by their unfamiliarity with the learning system. Research also reports a lack of peer interaction and student-teacher interaction online, which are both crucial for learning (Kamble et al., 2021). Furthermore, distance learning can also be a challenge for students who study academic subjects that require lab work (Dhawan, 2020; Radha et al., 2020). These issues may influence students’ academic outcomes and overall experiences (Omar et al., 2021).

### 2.2. Opportunities for online teaching

Despite the challenges, the remote-learning environment has also brought about new educational opportunities (Hoss et al., 2022; Li et al., 2022). For example, Azorín (2020) argues that the crisis provides scarce opportunities to trial, improve, and rethink the role, content, and innovative delivery methods of education. Meanwhile, Aguilera-Hermida (2020) believes that the remote-learning experience also promoted the development of digital literacy among students. Daniel (2020) argues that asynchronous learning, particularly suitable in digital formats, provides both teachers and students with flexibility and enables them to achieve a better balance between work and study. Moreover, online teaching also promotes flexibility while reducing expenses (Xhaferi and Xhaferi, 2020; Weldon et al., 2021). In addition to flexibility, researchers argue that online platforms and technology applications provide students with additional resources (Pokhrel and Chhetri, 2021; Sbaffi and Zhao, 2022) and enhance student academic achievements (Younas et al., 2022). Similarly, Fatoni et al. (2020) found that students can benefit from asynchronous materials (e.g., lecture recordings) to enhance their knowledge. There has also been an increase in the use of social media for sharing learning resources with students (Nasution et al., 2022). As Huang et al. (2021) point out, COVID-19 has created opportunities for universities and brings out innovative pedagogy and digital resources. However, it is uncertain how sustainable these benefits could be when universities start to transition back to ‘ordinariness’ and offer only face-to-face courses to students (Daniel, 2020).

### 2.3. Online to offline transition

Although COVID-19 is still spreading, coexistence with the virus has become an option for many countries, including the United Kingdom (Cabinet Office, 2022). Scholars, such as Hargreaves (2020), believe that temporary online education will move back towards the traditional campus-based model or a hybrid method. Meanwhile, Rashid and Yadav (2020) suggest that online teaching should remain an essential part of future teaching even when universities return to offline teaching.

In 2022, we have witnessed a shift to offline learning in the United Kingdom, as predicted by scholars (Daniel, 2020). As this is a new trend, there has been little research about the issues and challenges faced by students during this reverse transition. Among all university students, international learners suffer more challenges caused by external and transnational obstacles, such as border control, travel restrictions, flight cancellations, extra financial difficulties, and career issues (Hari et al., 2023). Therefore, this project will focus on the experience of a group of Chinese international students to explore their experiences during this transition.

## 3. Materials and methods

This research follows an interpretive paradigm and adopts qualitative research with an exploratory nature (Stebbins, 2001; Rudestam and Newton, 2014). It focuses on the experiences of Chinese international students during the online-offline transition to explore their perceptions and attitudes as well as the challenges they faced during this transition. Using a snowball sampling strategy, 24 semi-structured interviews were conducted with various cohorts of students, Campus Starters (*n* = 8), Remote Starters (*n* = 8), and Leave of Absence (LOA) students (*n* = 8). CS students began their on-campus life at the beginning of the first semester, which means they participated in more face-to-face courses than the other participant groups. While RS students travelled to the United Kingdom successively between Sep 2021 and Feb 2022, they engaged with both online and offline learning. Meanwhile, LOA students only experienced online learning and had to postpone their studies for a year. See Table 1 for detailed participant information.

**Table 1:** Participant information.

Participant ID	Gender	Degree	Duration of online learning	Type of study
1	Female	Master	1 week	Remote start
2	Female	Master	6 months	Remote start
3	Male	Master	N/A	Campus start
4	Male	Master	N/A	Campus start
5	Female	Bachelor	N/A	Campus start
6	Female	Master	6 months	Remote start
7	Female	Master	6 months	LOA
8	Male	Master	6 months	LOA
9	Female	Pre-Master	2 months	Remote start
10	Female	Bachelor	4 months	LOA
11	Female	Bachelor	6 months	LOA
12	Female	Master	N/A	Campus start
13	Female	Bachelor	6 months	LOA
14	Female	Master	6 months	LOA
15	Male	Master	6 months	LOA
16	Male	PhD	N/A	Campus start
17	Female	Master	N/A	Campus start
18	Male	Master	6 months	Remote start
19	Female	Master	6 months	Remote start
20	Female	Master	2 months	Remote start
21	Female	Master	6 months	LOA
22	Female	Master	8 months	Remote start
23	Male	Master	N/A	Campus start
24	Male	Master	N/A	Campus start

The interviews were conducted online with the help of meeting tools (Google Meet and Tencent Meeting) that enabled Chinese international students outside of the United Kingdom to participate in the interview. Due to the nature of this study, the research was conducted in two languages: English and Chinese (Mandarin). A pilot study was conducted prior to data collection to refine and revise interview questions and enhance the reliability of the research. The interview questions were divided into two sections. As part of the first section, we asked questions about student programmes, study durations, and previous transitional experiences. Depending on students’ learning modes (e.g., CS, RS or LOA), the second section contains different questions. Common questions focused on students’ decision-making when choosing different learning modes, their overall feeling towards the university’s online to offline transition, barriers they faced, specific events or activities that impacted their learning experiences, and their recommendations to the university. A design of the interview questions was submitted as Supplementary material.

The data from the semi-structured interview was then transcribed, translated, and analysed using a thematic analysis approach, which included becoming familiar with the data, generating initial codes, searching for themes, reviewing themes, defining themes, and producing a write-up (Braun and Clarke, 2006). This method is particularly suitable when analysing human experiences and perceptions, which also fits well with the objectives of this article. Specifically, informed by Kiger and Varpio (2020), our data analysis process involved the following steps: (1) familiarise all the interview transcripts and generate a general understanding of the dataset, (2) analyse each transcript in detail and note the initial codes according to the research questions, (3) examine all the initial codes and search for core themes, (4) reviewing the themes, interpreting their interrelationships, and answering the research questions, (5) defining and naming all the themes, identifying the issues, and making suggestions and (6) producing the report based on the themes.

The study received ethics approval from the university of [anonymised]. Informed consent was collected prior to the interviews. In addition, participants were informed of their right to withdraw from the study. The names of all participants have been anonymised. An ethics approval letter can be found in the Supplementary materials.

## 4. Results

Our interview data revealed a number of challenges that students faced during the online to offline transition at the university, namely policy, infrastructure, academic and financial challenges.

### 4.1. Policy-related challenges

Policy issues create enormous difficulties for all types of students (CS, RS and LOA). The identified policy challenges consist of two sub-themes: policy constraints and policy conflicts.

#### 4.1.1. Policy constraints

In the context of the COVID pandemic, policy constraints come from national-level travel restrictions and institutional-level teaching guidelines. Travel restrictions were enormous challenges for international students. During the transition, the RS and LOA students suffered from local lockdowns and strict flight controls, which forced them to be separated from the offline-learning environment. These issues were often unpredictable and left students with limited time to respond. One RS student said that:

After my flight was suspended, I couldn’t come in time and had to buy a new ticket. This was a bit troublesome. The travel procedures are quite complicated. If I didn’t do it in advance, I couldn’t arrive at the expected time. (Participant 6, RS, PGT)

The travel restrictions imposed a significant influence on students’ choices of teaching approaches. Students were unable to shorten their physical distance from campus. Therefore, students had to choose from the available options. In other words, policy constraints deprived them of the opportunity for campus learning. One LOA student complained that:

I was faced with various quarantine policies, […] according to the domestic policy, there was no way for me to take public transportation, let alone apply for a visa to study abroad. (Participant 7, LOA, PGT)

Similarly, when asked about the reason for making the online choice, an RS student answered by saying that:

My flight was suspended at that time. But actually, I wanted to come over. (Participant 6, RS, PGT)

In addition, some students expressed concerns about the challenges they may face after their studies in the United Kingdom were completed. To avoid quarantine upon their return to China, students were hesitant or even resisted the offline shift. One bachelor’s student who was unwilling to transition said that:

[…] if I go to the UK, I need to be prepared to not go home for the next two or three years, because China’s strict policy makes it very hard to return home, which I couldn’t accept at that time. (Participant 11, LOA, UG)

A master’s student who postponed the journey to the United Kingdom shared a similar concern:

Although the pandemic-management policy in Britain allows for total freedom in 2022, in my region, pandemic control is particularly strict. […] If I got the coronavirus, it would be very difficult for me to return home, where I would face a long quarantine. So, I didn’t want to come to the UK at that time. (Participant 6, RS, PGT)

Apart from their choice of learning methods and travel concerns, the travel restrictions imposed psychological and financial pressure on students, discouraging them from moving to offline learning. For example, one LOA student expressed that:

[…] There were moments that I wished to go to the UK for on-campus learning. However, the fight tickets were very expensive, which led to a lot of pressure. […] My flight could easily have been suspended and triggered the circuit cancel policy. […] It ultimately made me anxious about the delay in my studies. […] Everyone was extremely anxious. (Participant 15, LOA, PGT)

The institutional-level teaching guideline is another major challenge. The guideline refers to the mandatory offline-learning policy, which the university created without providing prior notice to students. This led to considerable impacts for RS and LOA students. This sudden policy shift also made it imperative for students to prepare for the transition in a short period of time. Data shows that the sudden change demotivated many students from transiting to offline learning. One LOA student said:

I thought that they should have included online classes as an option in the second semester since the school had made such an inquiry. […] So, I didn’t have, or consider to have my visa, accommodations, and so on prepared. (Participant 11, LOA, UG)

Another identified impact of the shift is the adjustment. This is primarily related to RS students. Universities in the United Kingdom have had resources in place for years to support students’ orientation at the beginning of the term. Therefore, CS students received sufficient support when familiarising themselves with offline teaching and the learning environments (e.g., campus facilities). Arriving at the campus halfway through the term meant that RS students needed to spend time and effort adapting to the new environment with less university support compared to CS students. As RS students had to make last-minute arrangements, travel expenses and overseas living costs were sometimes exceptionally high.

Online classes are […] very convenient, which saves both time and money. […] When I first came here, I had to adapt to the life here […] which might take me a week to adapt, […] The transition period of that week will affect my courses, which is quite challenging. (Participant 6, RS, PGT)

#### 4.1.2. Policy conflicts

Conflicts exist between travel restrictions and teaching guidelines. Specifically, the university policy requires face-to-face teaching, but the travel policy imposes many restrictions both in China and in the United Kingdom. This mismatch created a practical challenge for international students, especially those in the LOA and RS groups. Some LOA students were under a local lockdown and were forced to take a leave of absence. One LOA student said:

I was forced to make that choice (leave of absence), […] Many remote students were forced to go there and study on campus because they didn’t want to take a break from college. We were forced to do that. (Participant 11, LOA, UG)

The conflicts between teaching guidelines and quarantine policy in the United Kingdom at the time were also challenging for certain CS students. The quarantine policy blocked face-to-face registration and sessions despite the arrival of CS students in the United Kingdom. The university also prevented them from attending online sessions as they were registered as CS students. One CS student said:

When I first came to the UK, I was required to stay in quarantine for 14 days. However, I couldn’t attend any classes in the Blackboard system until I completed registration offline to receive my student card. […] This resulted in me missing some of my classes. (Participant 3, CS, PGT)

Conflicts also exist in policies between the university and academic departments. For example, the university provided remote-teaching alternatives during the first semester, but some schools or modules only offered offline exams, resulting in students taking a leave of absence. One LOA student claimed that:

It was quite sudden that we were told to attend offline exams while students of other majors could take their exams online. We thought it was unfair. I even thought the school was way out of line. […] I got 0 on that module and need to retest. […] I could have received a good result after studying for so long. […] I have to go over there next September and start my sophomore year all over again, which is a huge waste of time. (Participant 10, LOA, UG)

In addition to study interruptions and exam delays, students also reported that the sudden transition negatively impacted their career planning and psychology. The interruption of studies has necessitated postponing the original study work plan and introduced uncertainty. The fear of further uncertainty and potentially needing to take a gap year has created additional anxiety for LOA students. The following arguments support these identified impacts:

My plan was disrupted! My plans for my studies and future career were completely messed up. And there was also a big impact on my life. I was at home by myself during the lockdown. Plus, the leave of absence stuff, I was depressed and not in a good mood […] I couldn’t help feeling anxious at night. (Participant 14, LOA, PGT)

Due to a year of delay, I have concerns in terms of time. Also, I have age anxiety. (Participant 13, LOA, UG)

### 4.2. Infrastructure challenges

#### 4.2.1. Technology constraint

Technical issues mainly occurred during the first half of the academic term. The university incorporated live-streaming technologies, which allow for face-to-face teaching to be broadcasted to remote students. All the international students experience some online components in the autumn semester, such as lecture recordings and asynchronous materials. Therefore, this paper infers that technical constraints are common challenges for all student groups. Concretely, the technical challenges manifest in unstable networks, unclear audio, and blurry pictures in the live class. These factors mainly affect the synchronisation and efficiency of the live-streaming sessions. When describing technical issues, students said:

The problem was the network latency. […] Occasionally, it was very laggy. (Participant 1, RS, PGT)

Sometimes, there is noise in the livestream classes and recordings. This is inevitably with lots of students in a classroom. (Participant 4, CS, PGT)

I need to use a VPN to attend classes, which can be troublesome and lags sporadically. (Participant 10, LOA, UT-Year2)

Students considered these technical issues to be temporary. Therefore, their overall learning outcomes and experiences were not hugely impacted. When asked about this, students reported that:

It wasn’t too bad. […] I am satisfied with my studies during those six months. (Participant 8, LOA, PGT)

I was quite content with my learning results. (Participant 15, LOA, PGT)

#### 4.2.2. Campus facility constraint

The challenges caused by the inconvenience of campus facilities mainly affect RS students who newly completed the transition. After experiencing online learning, RS students needed to readjust to offline classrooms. Compared to CS students, the campus is new to them. Furthermore, there was no orientation week to support students who were unfamiliar with the campus. This unfamiliarity made them feel that the campus facilities were not easy to access. For example, one student explained:

I couldn’t find the classroom on my first day on campus. […] The classroom was so far away from my apartment and located on the second floor of a basement. Twisting and winding, it was like a maze. […] I looked for it for a long time. (Participant 1, RS, PGT)

The facility constraint impacts learning convenience. Preference for convenience makes students reluctant to accept new changes. Therefore, the physical inconvenience of offline facilities and a lack of university support regarding induction made them more unwilling to give up flexibility and easy access to teaching. This is one of the reasons for the resistance to the transition. One student claimed:

Before the transition, I could sit at home and have classes without running to the campus. […] The campus of my university is quite big, so it was easy to get lost. (Participant 5, CS, UG)

### 4.3. Academic challenges

#### 4.3.1. Reduction of digital support

Online teaching facilitates a wealth of digital learning tools and can promote students’ digital literacy skills. Apart from some efficient and innovative platforms, including learning management systems (e.g., Blackboard), Cloud-based collaboration tools (e.g., Google), and social media (e.g., YouTube), digital tools, such as recording software, auto-captions, and real-time translation, also played critical roles in improving academic results. Students were immersed in the digital environment for the first half of the academic term and were dependent on the digital tools that supported them in online learning. When they fully transitioned to offline learning, many digital tools became unavailable, which created a massive challenge for students. Almost all RS and CS participants stressed the importance of some digital tools for their learning. Among these tools, the course recording and language assistance software were most valuable to students. Course videos allow students to revisit lectures, while translation tools help them overcome language barriers, contributing to enhanced learning outcomes. Furthermore, international students rely heavily on captions and auto-translation to overcome language barriers and achieve academic progress. According to multiple students:

I could turn to the recordings at any time, according to my learning progress, if there was anything I didn’t figure out or I had missed in offline classes. […] In the first semester […] I relied heavily on the recordings and slides of the classes. (Participant 12, CS, PGT)

I could turn to the subtitles when I couldn’t understand the lecture. […] I was able to understand more content in class compared with the second semester. (Participant 2, RS, PGT)

The learning experience is better with online lectures as there are auto-captions that we can use. (Participant 18, RS, PGT)

The chatbot feature allows the tutor to answer all questions one by one. This can benefit all students. Offline lectures can become crowded when students are asking questions. Tutors may not be able to address all of them. (Participant 24, CS, PGT)

Students’ reliance on digital tools is directly linked to their learning experiences and outcomes. This reliance can also be arguably linked to students’ resistance to offline learning. Face-to-face learning becomes more challenging if students experience language barriers. As one student argued:

I became reliant on the subtitles when listening to online classes. I would habitually look at the subtitles and not think about what the teacher or my classmates were saying. And, when I switched to on-campus learning, I would find it hard to adapt in terms of listening and other aspects. (Participant 1, RS, PGT)

Students value the digital skills they have developed through online education. Our data suggests that online learning initiates innovative approaches and facilitates the utilisation of technology, such as digital assistance tools, online cooperation software, social media platforms, etc. These innovative and beneficial approaches also enhance the digital skills of students, which is indispensable in the Information Age. Some participants believe that the enhancements brought about by online learning contribute to their future development:

The informative approach will become a major trend in the future. For instance, working from home or something like this. […] it would be of great help if you have experience in online classes and are very skilled and seasoned in online information processing, operation, and learning. Because I worked as an intern during the LOA period and there were often online meetings or communication. […] My previous online experience was valuable during my internship. (Participant 11, LOA, UG).

However, the transition to offline learning has significantly decreased the use of digital tools and materials for learning and teaching. Hence, reliance on digital support also drove some LOA students away from offline learning. One LOA student conveyed the following view:

I can refer to the recordings, […] It helps with the absorption and consolidation of knowledge. […] Is it only in offline classes that you can learn well? Not necessarily. (Participant 14, LOA, PGT)

This challenge also affects the frequency and timing of students’ utilisation of IT tools and platforms. For example, with the total shift back to offline learning, the recording and captioning tools are no longer available. Students’ time spent on digital resources, such as E-Library and Blackboard, was also reduced.

I spent more time on Blackboard and reading course materials. […] I’m more frequently using online tools. […] Then, during the second semester, […] I didn’t log on to Blackboard very often. (Participant 2, RS, PGT)

#### 4.3.2. Reduced flexibility

Flexibility is a unique feature of online learning. This term describes flexibility in time and location, which could contribute to crisis resilience. Therefore, many students, after experiencing online learning, were reluctant to give up flexibility in teaching. Specifically, the recorded and asynchronous materials allowed students to learn more flexibly. Furthermore, these factors ensure that teaching is unaffected by external contingencies, such as the strike movement that occurred several times during the academic year.

Last semester, […] I could just turn on my computer and attend my classes, […] when I came here, I had to walk a long distance to take the courses, which I found quite tiresome and time-consuming. (Participant 2, RS, PGT)

I wasted my time running to the classroom, which ended up being empty (due to staff strikes), […] The same thing could happen online, but it probably would not affect me as much. (Participant 3, CS, PGT)

Another reason is related to the previously identified factor: digital support. Lecture contents are easier to understand for international students with the help of digital tools. In addition, after transitioning to offline learning, students need to spend more time and effort on self-study to make up for the information missed during face-to-face lectures due to language barriers. One student points out:

Because there is no recording, […] I need to listen attentively, […] I need to spend more time and energy, such as looking at the courseware again, or reading a lot of relevant materials to make up for some knowledge I missed in class. (Participant 5, CS, UG)

### 4.4. Financial challenges

The transition from online to offline also increased the total financial cost of education. This issue posed different levels of challenges depending on the cohort of students. For RS and LOA students, the increased cost is significant, especially in the context of COVID. The sudden transition means that students must spend a lot of money on living costs, accommodations, international flights, and COVID-related expenses. One LOA felt particularly stressed and complained by saying:

A round trip ticket is very expensive, nearly £10,000. It is not a small amount of money for my family. […] It is hard for me to make a casual choice. (Participant 15, LOA, PGT)

Due to the lockdown, there was a delay in the postal service. As a result, I didn’t get my visa and passport returned to me on time, and missed my flight as a result. There was no refund. I had to buy a new flight ticket. (Participant 22, RS, PGT)

These additional significant financial costs led to psychological stress for students and may have led to their decision to postpone their studies. Completing a degree at the minimum possible cost is very attractive to many students. Therefore, the cost challenge is a fundamental factor that drives their reluctance to transition.

For international students, it requires a huge amount of money to live and study in another city. For those who have one year course, the online option can actually help them save a lot of money for their families. (Participant 11, LOA, UG)

The impact on CS students is less severe. The fact that they were already involved in offline classes means that they had considered the issue of education expenditure and made a choice with sufficient time to plan the trip. The additional high cost, due to COVID-19, is within their expectations.

## 5. Discussion

This research developed a nuanced understanding of the challenges that influence Chinese international students during the online-to-offline transition. Several challenges these students faced during the transition were identified, including policy issues, infrastructure constraints, academic barriers, and financial burdens. Among all the identified challenges, policy challenges posed the most serious issues for international students, and the impacts were far more severe than those brought about by other challenges. Issues such as academic, social and infrastructure only impact the transition’s quality. In contrast, policy issues, especially policy conflicts, may determine the success or failure of some students’ transitions. The emergence of LOA students represents the failure of their campus-oriented shift. The findings evidenced that LOA students were the most vulnerable among all students, as this group faced interruptions in their studies, delays in career planning, and enormous psychological pressure. This accords the finding of Sahu (2020), who suggests that policies, such as travel restrictions bring a series of secondary challenges, including delays in examinations, monetary problems, admission issues, study interruption, psychological health, and career plans.

All participants displayed resistance to a complete transition from online to offline learning. However, the resistance was more evident among the RS and LOA groups. This could be because these groups of students were unwilling to accept the challenges brought upon them by a sudden shift to offline learning. Another noticeable and common reason for resistance is the reliance on digital tools. Online learning tools are most effective in assisting with language barriers. With the help of recording, captioning, and live translation tools, participants reported significantly improved learning outcomes in lectures and eventually became reliant on them (Fatoni et al., 2020; Pokhrel and Chhetri, 2021). This is consistent with the finding of Weldon et al. (2021), who argue that lecture recordings significantly benefit students during the revision and assessment stages. Moreover, many efficient and innovative technologies were introduced during the online period, such as screensharing during group discussions or using learning materials from social media. Students also benefit from more frequent use of digital tools, as their digital skills are enhanced. This finding accords with the research conducted by Aguilera-Hermida (2020), who argues that the online-oriented transition promotes the utilisation of technology and digital literacy. Similarly, Noor et al. (2022) also highlights the benefits of information and computer technology (ICT) skills development for students as a result of a rapid growth of technology in education during the pandemic.

Based on the findings, this research has the following recommendations. This research suggests that a complete and sudden switch to offline teaching is not a perfect solution. Advance notice should be given to students at the offer stage rather than forcing a sudden change upon students midway through their studies. Lei and So (2021) argue that students are sensitive to the sudden switch between different learning modalities. In addition to considering national policies, universities in the United Kingdom should also take into account international policies that may affect international students’ travels and studies. In addition, universities should implement a consistent policy across all academic departments to minimise the impact on students who study for joint degrees or attend modules offered by more than one department. In addition, the value of digital tools and resources should be acknowledged and sustained to enhance the student learning experience, academic outcomes, and the development of digital literacy. Our finding supports the view of previous research, which highlights the value of a blended teaching approach and sustained digital resources for teaching in a post-pandemic age (Hargreaves, 2020; Rashid and Yadav, 2020). Finally, universities should consider enhancing their resources to support the transition of those students returning from a leave of absence. Students from this group are often the most vulnerable and underrepresented. In the current system, students are most likely to receive support during the orientation or induction week. Dedicated support staff or resources could be made available for students who miss induction weeks, such as late arrivals and students who return from a leave of absence.

## 6. Conclusion

This research aims to study the challenges encountered by international students during the reverse transition from online to offline teaching in the post-pandemic era. The findings identified challenges faced by Chinese international students during this transition, namely, policy issues, infrastructure constraints, academic barriers, and financial burdens. First, the policy issues are most challenging for students resulting in a broad range of negative impacts. Our data suggests that LOA students are the most vulnerable to study interruptions, career plan disruptions, and psychological pressure. More support should be provided to this cohort when they resume their studies. Second, a consistent teaching method across the academic year is most valued by students. Almost all participants displayed negativity and resistance towards a sudden move to offline learning. Sudden changes in teaching can cause considerable disruptions to students. Thus, advanced notice should be provided to students if such sudden changes are unavoidable. Third, the complete reversion to the traditional face-to-face model is not a perfect option. Online modality has unique and irreplaceable benefits, promoting innovation and enhancing students’ digital skills. Universities should retain the digital resources and pedagogy developed during the pandemic and incorporate them into offline teaching.

This research also presents several limitations. Due to the research scope, the subject is limited to Chinese international students. Particular challenges brought about by the policies in China are not representative of the entire international student community. Future studies may select a broader range of subjects. Further studies could also look at the readjustment issues faced by LOA students who have been found to be most vulnerable in this research. In addition, the perception of the participants may change over time. A longitudinal study is needed to determine the long-term impact of the transition on students.

## Data availability statement

The raw data supporting the conclusions of this article will be made available by the authors, without undue reservation.

## Ethics statement

The studies involving human participants were reviewed and approved by University of Sheffield Information School Ethics Committee. The patients/participants provided their written informed consent to participate in this study.

## Author contributions

All authors listed have made a substantial, direct and intellectual contribution to the work, and approved it for publication.

## Conflict of interest

The authors declare that the research was conducted in the absence of any commercial or financial relationships that could be construed as a potential conflict of interest.

## Publisher’s note

All claims expressed in this article are solely those of the authors and do not necessarily represent those of their affiliated organizations, or those of the publisher, the editors and the reviewers. Any product that may be evaluated in this article, or claim that may be made by its manufacturer, is not guaranteed or endorsed by the publisher.
