# Supplementary material for: From online to offline education in the post-pandemic era: Challenges encountered by international students at British universities
Source: Front Psychol. 2023 Jan 18;13:1093475. doi: 10.3389/fpsyg.2022.1093475 (PMC9890062; doi:10.3389/fpsyg.2022.1093475)
Supplement: Supplementary file 1 [file Data_Sheet_1.docx]

Interview Questions

Basic question:

~What is your programme level?

~What is the duration of your programme?

~What is your study path during this transition? (Did you participate in offline learning from the start of the academic year 2021/2022? Did you return to campus after a period of online learning? Did you choose the leave of absence in the spring semester? )

~Have you ever had an online or offline learning experience before? If you have, could you please share your previous experience?

Detail question (CS Student):

~ Why did you make the decision to choose campus learning from the beginning?

~ Did all your education activities take place on campus?

~ If not, what kinds of online activities did you participate in? (e.g., lecture, seminar, tutorial, group work, exam)

~ During the offline learning period, what is your overall feeling about offline education? ~ Can you talk about some pros & cons of the online activities during campus-based learning?

~ What kind of challenges did you face when switching frequently between online and offline education activities? (You can talk about this question from social, academic, emotional, or any relevant aspects)

~How did these challenges impact your learning outcomes and experience?

~How did you adapt to the transition and overcome these challenges?

~ Could you please talk about one of the most significant things that positively influence your learning experience during the transition?

~Could you please talk about one of the most significant things that negatively influence your learning experience during the transition?

~ What kinds of learning activities or academic support provide you with the best learning experience during this transition? (lecture, seminar, group work, etc.)

~ From your opinion, which education type benefits you most? (online, hybrid or offline)

~ What support services do you need most?

Detail question (RS students):

~ How long have you been a remote learner?

~ What reasons make you become a remote learner?

~ During the online learning period, what is your overall feeling about online education? ~ Can you talk about some pros & cons of online education?

~ Why did you make the decision to back your campus learning?

~ What kind of challenges did you face when you move back to the campus education environment? (You can talk about this question from social, academic, emotional, travel, or any relevant aspects)

~How did these challenges impact your learning outcomes and experience?

~How did you adapt to the transition and overcome these challenges?

~ Could you please talk about one of the most significant things that positively influence your learning experience during the transition?

~Could you please talk about one of the most significant things that negatively influence your learning experience during the transition?

~ What kinds of learning activities or academic support provide you with the best learning experience during this transition? (lecture, seminar, group work, etc.)

~ From your opinion, which education type benefits you most? (online, hybrid or offline)

~ What support services do you need most?

Detail question (LOA Students):

~ How long have you been a remote learner?

~ What reasons make you become a remote learner?

~ During the online learning period, what is your overall feeling about online education? ~ Can you talk about some pros & cons of online education?

~ Why did you resist backing your campus leaning?

~ What kind of challenges did you face during online learning? (You can talk about this question from social, academic, emotional, travel, or any relevant aspects)

~ How did these challenges impact your learning outcomes and experience?

~ How did you overcome these challenges?

~ Could you please talk about one of the most significant things that positively influence your learning experience during online learning?

~Could you please talk about one of the most significant things that negatively influence your learning experience during online learning?

~ Why did you make the decision to choose ‘leave of absence?

~ How do you think the leave of absence would impact your study? What are the pros and cons?

~Are you looking forward to coming back to study? What concerns do you have when you finish the leave of absence and come back to study?

~ What is your plan for this year? (Find a job, self-study or relax)

~ When you continue your studies, how would you like to be taught and why? (online, hybrid or offline)

~ What support services do you need most?
